# Author Correction: Global subsidence of river deltas

**DOI:** 10.1038/s41586-026-10294-0

**Published:** 2026-02-24

**Authors:** L. O. Ohenhen, M. Shirzaei, J. L. Davis, A. Tiwari, R. Nicholls, O. Dasho, N. Sadhasivam, K. Seeger, S. Werth, A. J. Chadwick, F. Onyike, J. Lucy, C. Atkins, S. Daramola, A. Ankamah, P. S. J. Minderhoud, J. Oelsmann, G. C. Yemele

**Affiliations:** 1 Department of Earth System Science, University of California, Irvine, Irvine, CA USA; 2 Department of Geosciences, Virginia Tech, Blacksburg, VA USA; 3 Institute for Water, Environment and Health, United Nations University, Richmond Hill, Ontario Canada; 4 Lamont-Doherty Earth Observatory, Columbia University, New York, NY USA; 5 Texas A&M AgriLife Research Center, Corpus Christi, TX USA; 6 Tyndall Centre for Climate Change Research, University of East Anglia, Norwich, UK; 7 School of Engineering, University of Southampton, Southampton, UK; 8 Institute of Geography, University of Cologne, Cologne, Germany; 9 Soil Geography and Landscape Group, Wageningen University and Research, Wageningen, The Netherlands; 10 Department of Civil and Environmental Engineering, Virginia Tech, Blacksburg, VA USA; 11 Department of Civil, Environmental and Architectural Engineering, University of Padova, Padova, Italy; 12 Department of Groundwater and Water Security, Deltares Research Institute, Utrecht, The Netherlands; 13 Department of River-Coastal Science and Engineering, Tulane University, New Orleans, LA USA

**Keywords:** Natural hazards, Environmental impact, Geophysics

Correction to: *Nature* 10.1038/s41586-025-09928-6 Published online 14 January 2026

In the version of the article initially published, J. Oelsmann’s surname appeared incorrectly (as Olsemann) and has now been amended in the HTML and PDF versions of the article.

