# Supplementary material for: Global subsidence of river deltas
Source: Nature. 2026 Jan 14;649(8098):894–901. doi: 10.1038/s41586-025-09928-6 (PMC12823437; doi:10.1038/s41586-025-09928-6)
Supplement: Supplementary file 1 — Supplementary Figs. 1–8. [file 41586_2025_9928_MOESM1_ESM.pdf]

nature portfolio

https://doi.org/10.1038/s41586-025-09928-6

Supplementary information

Global subsidence of river deltas

In the format provided by the authors and unedited

Supplementary Materials for

Global Subsidence of River Deltas

Leonard O. Ohenhen, Manoochehr Shirzaei, James Davis, Ashutosh Tiwari, Robert J. Nicholls, Oluwaseyi Dasho, Nitheshnirmal Sadhasivam, Katharina Seeger, Susanna Werth, Austin Chadwick, Florence Onyike, Jonathan Lucy, Carmen Atkins, Samuel Daramola, Alexander Ankamah, Philip S. Minderhoud, Julius Oelsmann, Gergino C. Yemele

Corresponding author: oohenhen@uci.edu

The PDF file includes:

Supplementary Figs. 1 to 8
Supplementary Tables 1 to 3

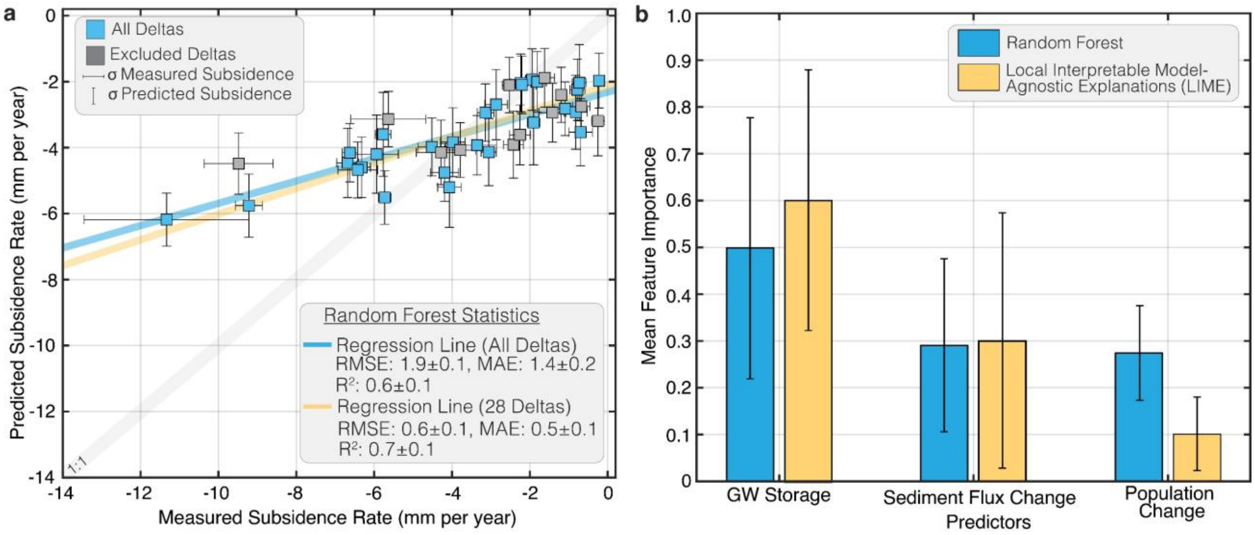

Fig. 1. Random Forest (RF) Analysis of Subsidence in 40 Deltas. (a) Scatter plot of predicted versus measured subsidence rate (mm per year) derived from the RF model. Gray squares represent deltas excluded due to low Local Interpretable Model-Agnostic Explanations (LIME) fidelity ($R^2 < 0.5$). **(b)** RF- and LIME-derived feature importance for predictors (groundwater storage, sediment flux change, and urban fraction change).

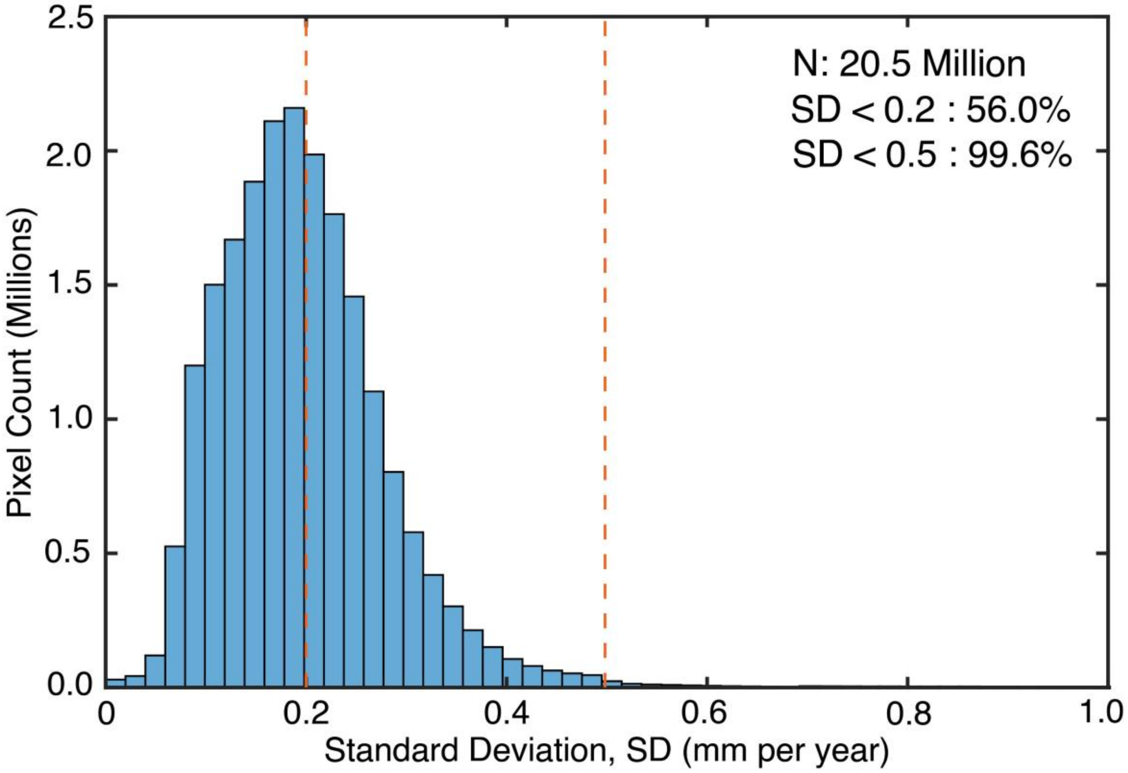

Fig. 2. Distribution of Standard Deviation (SD) across the 40 Deltas. Histogram showing the distribution of SD (mm per year). The dashed vertical lines indicate the SD thresholds of 0.2 mm per year and 0.5 mm per year. N is the total number of pixels across all deltas.

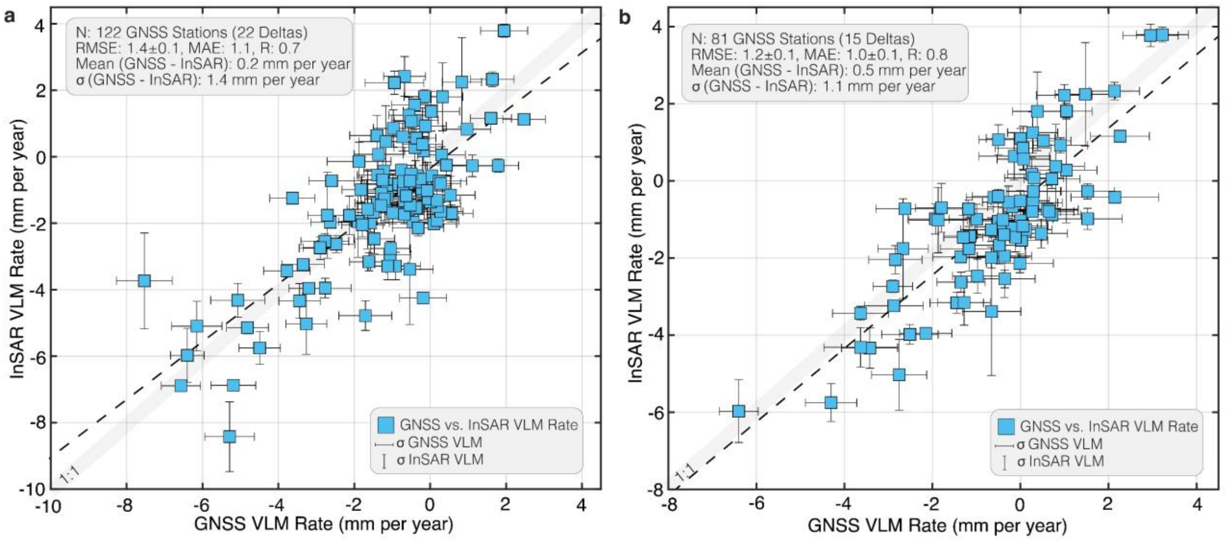

Fig. 3. Validation of the Vertical Land Motion (VLM) rates across the 40 Deltas. Bivariate plot comparing global navigation satellite system (GNSS) VLM rates with interferometric synthetic aperture radar (InSAR)-derived VLM rates using **(a)** historical long-term GNSS records, and **(b)** InSAR observation period (2014 – 2023).

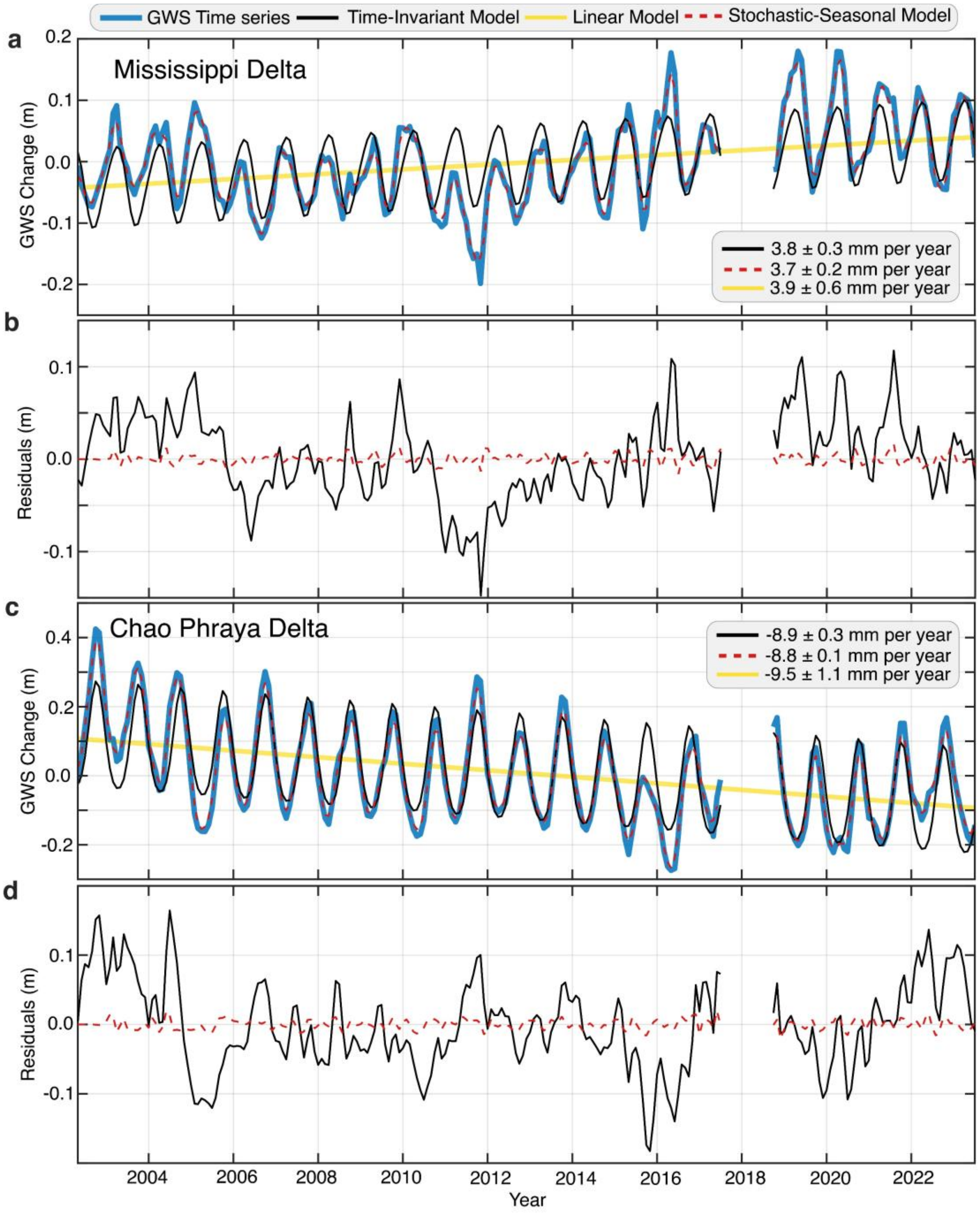

Fig. 4. Comparison of Time-Invariant and Stochastic-Seasonal Models for Groundwater Storage Trends. Time series of Gravity Recovery and Climate Experiment (GRACE)-derived groundwater storage (blue lines) for the **(a)** Mississippi and **(c)** Chao Phraya deltas, along with the time-invariant (black lines), linear (yellow lines), and stochastic-seasonal (red dashed lines) models. Residuals of the time-invariant and stochastic model for the **(b)** Mississippi and **(d)** Chao Phraya deltas.

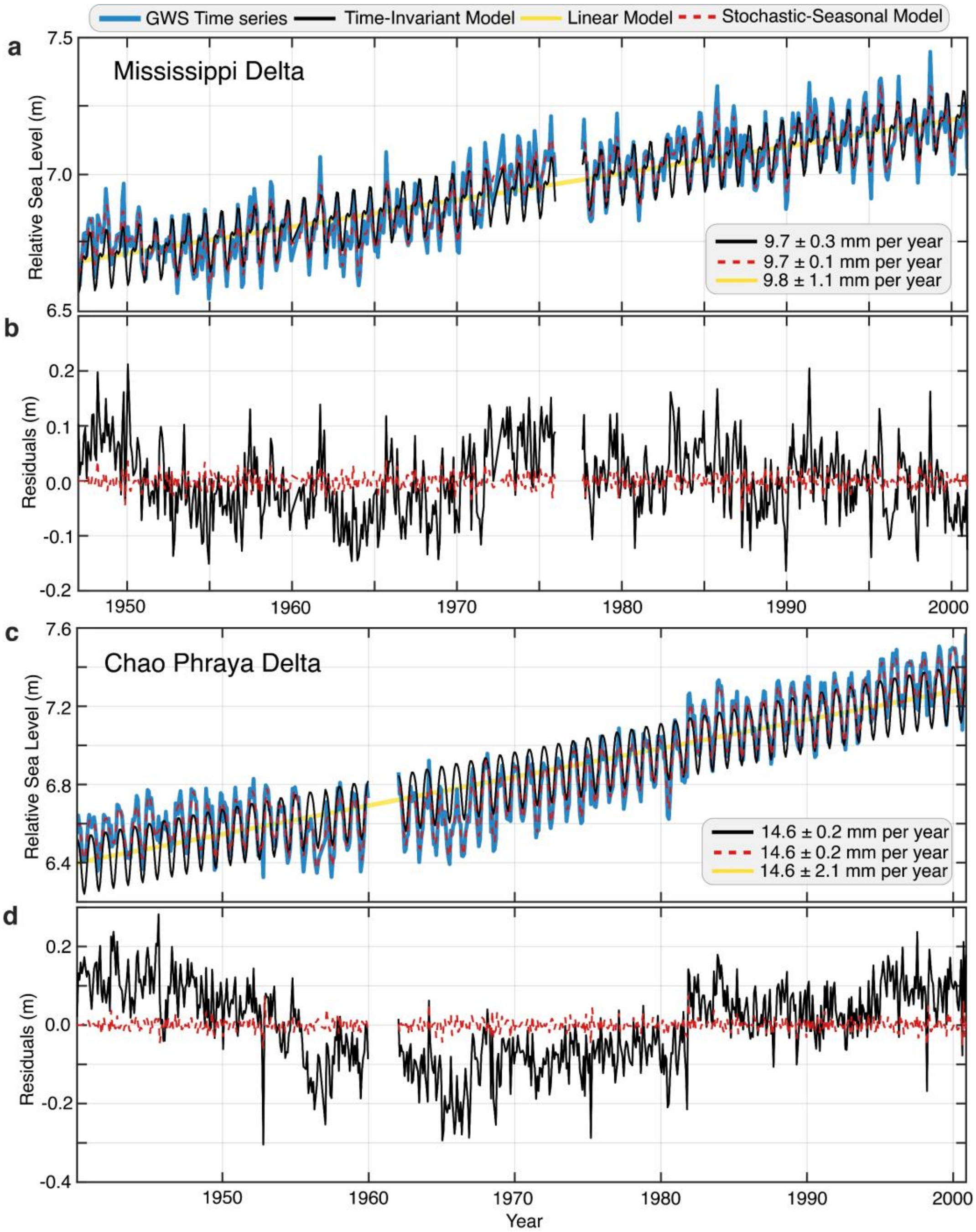

Fig. 5. Comparison of Time-Invariant and Stochastic-Seasonal Models for Sea Level Trends. Time series of 20th century relative sea level changes from tide gauges (blue lines) for the **(a)** Mississippi and **(c)** Chao Phraya deltas, along with the time-invariant (black lines), linear (yellow lines), and stochastic-seasonal (red dashed lines) models. Residuals of the time-invariant and stochastic model for the **(b)** Mississippi and **(d)** Chao Phraya deltas.

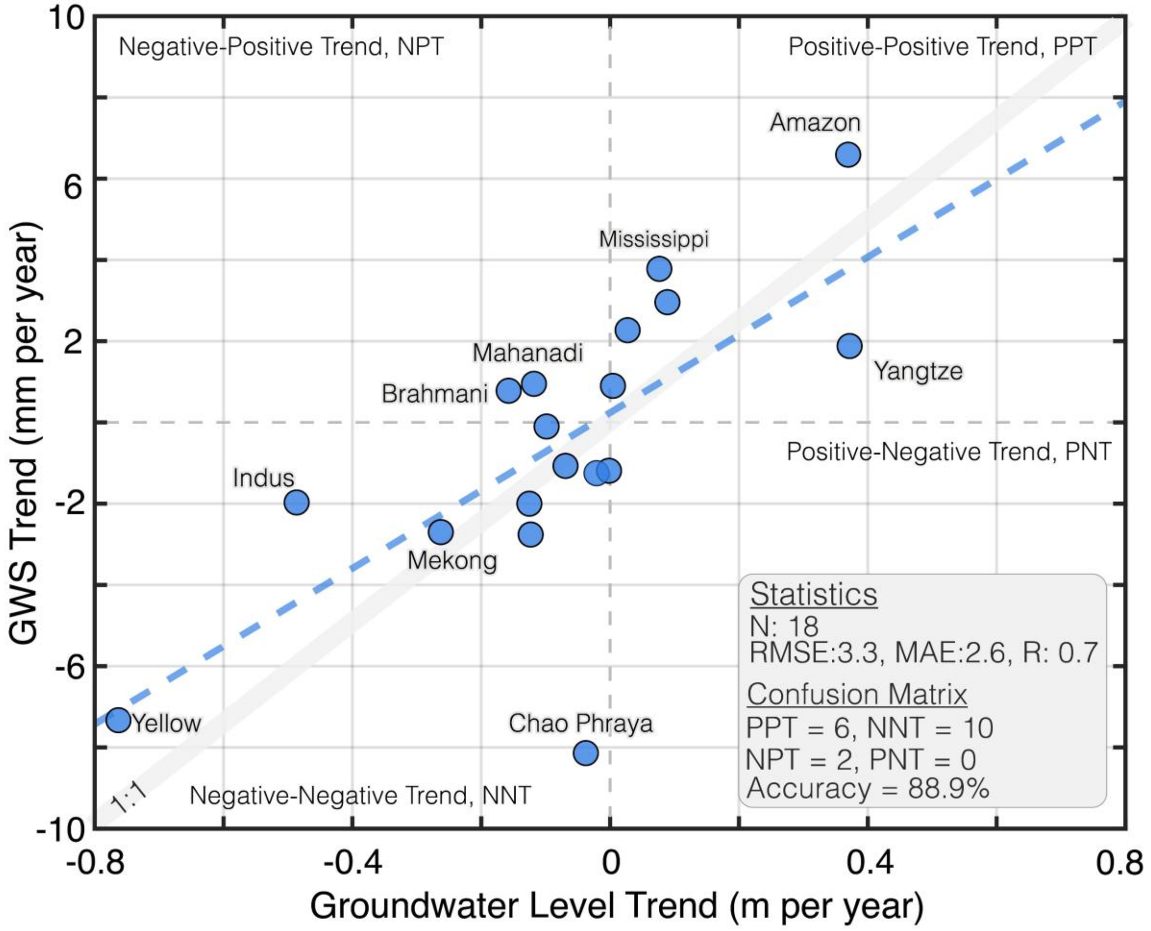

Fig. 6. Validation of GRACE-Derived Groundwater Storage (GWS) Trends. Comparison of GRACE-based GWS trends (y-axis) and in-situ groundwater level trends (x-axis) for 18 deltas. The 1:1 line (gray) represents perfect agreement, and the dashed blue line is the linear regression fit (R = 0.7; $R^2 = 0.5$). Trend agreement is summarized using a confusion matrix by categorical match in sign: positive-positive (PPT), negative-negative (NNT), and mismatched trends (NPT/PNT). Overall classification accuracy is 88.9%. Groundwater level data were obtained from 13 deltas in Jasechko et al. (2024) and 5 deltas from the Global Groundwater Monitoring Network database (IGRAC, 2024).

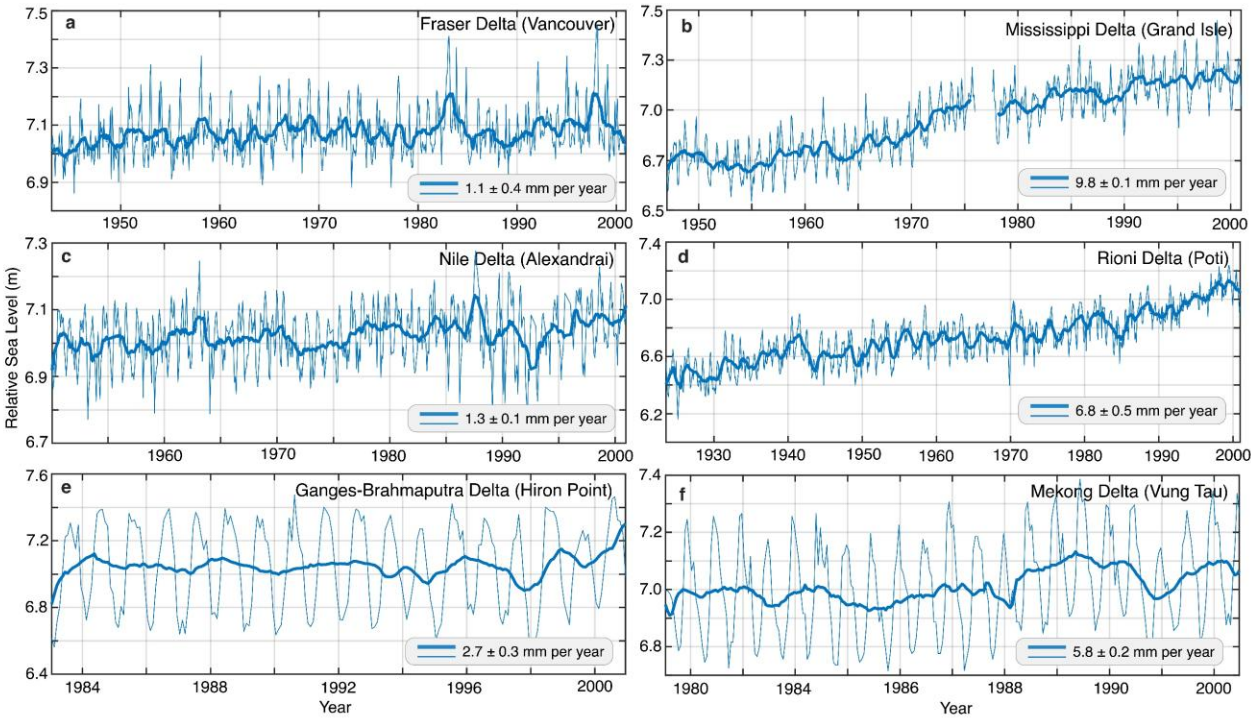

Fig. 7. Relative Sea Level (RSL) for the 20th Century for six Representative Deltas. Monthly (thin blue lines) and annual moving mean (thick solid blue lines) of RSL time series from tide gauges in the **(a)** Fraser, **(b)** Mississippi, **(c)** Nile, **(d)** Rioni, **(e)** Ganges-Brahmaputra, **(f)** Mekong deltas. The relative sea level rise rates for all 40 deltas are summarized in Supplementary Table 1.

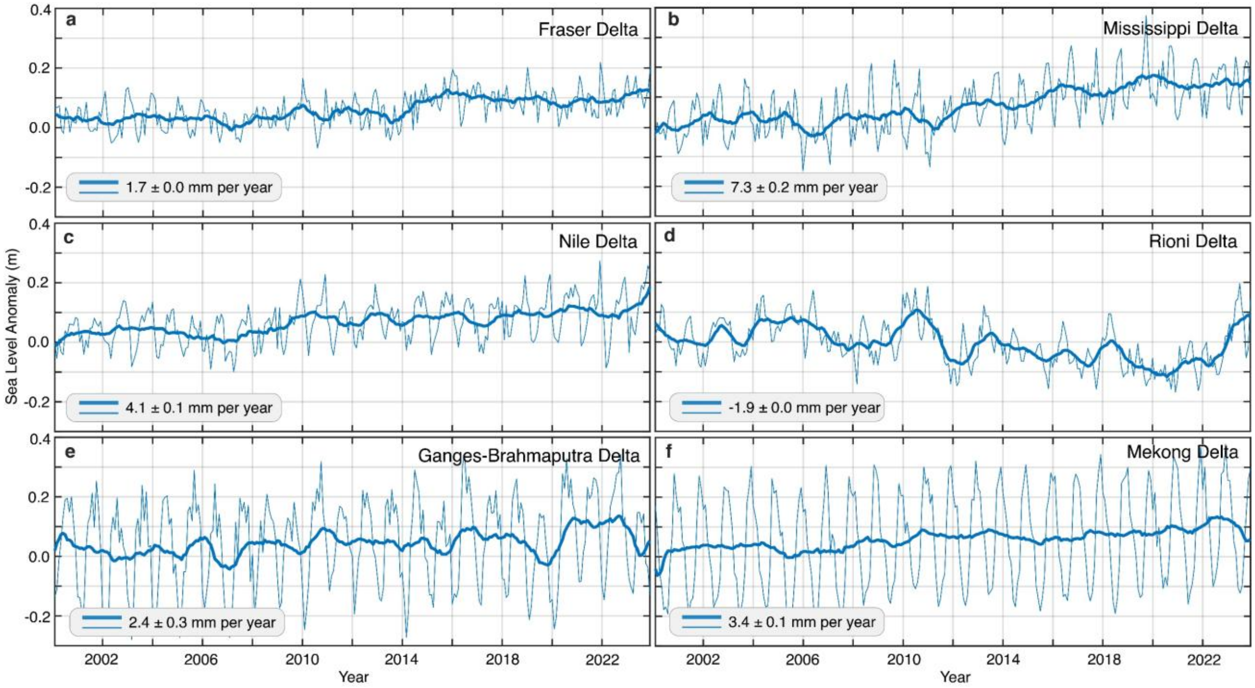

Fig. 8. Sea Level Anomaly (SLA) for the 21st Century for Six 6 Representative Deltas. Monthly (thin blue lines) and annual moving mean (thick solid blue lines) of SLA time series derived from satellite altimetry during 2001 to 2023 in the **(a)** Fraser, **(b)** Mississippi, **(c)** Nile, **(d)** Rioni, **(e)** Ganges-Brahmaputra, **(f)** Mekong deltas. The sea level rates for all 40 deltas are summarized in Supplementary Table 1.

Table 1. Land Subsidence, Sea Level Rise, Population, and the Notre Dame Global Adaptation Index (ND-GAIN) Adaptation Readiness Score for 40 Deltas. This table summarizes the dataset analyzed in this study, including land subsidence, sea level rise (historical, present, and projected), population (total and population on low elevation areas), and ND-GAIN adaptation readiness scores for each delta. Details on dataset sources and processing methodologies can be found in the Methodology and Data Availability sections.

<See attached Excel sheet>

Table 2. Anthropogenic Drivers of Subsidence in Deltas. This table summarizes the dataset for groundwater storage rate (mm per year), sediment flux, urban fraction, and the Local Interpretable Model-Agnostic Explanations (LIME)-derived feature importance of each predictor across all deltas.

<See attached Excel sheet>

Table 3. Synthetic Aperture Radar (SAR) Datasets for 40 Deltas. This table summarizes the processed orbits, paths, and frames for each delta. Ascending and descending orbits were processed for 27 deltas, while either ascending or descending acquisitions were processed for 13 deltas.

<See attached Excel sheet>
